# Breast Cancer Interaction Network Concept from Mostly Related Components

**DOI:** 10.31661/gmj.v8i0.1298

**Published:** 2019-08-07

**Authors:** Mostafa Rezaei-Tavirani, Mona Zamanian-Azodi, Davood Bashash, Naybali Ahmadi, Mohammad Rostami-Nejad

**Affiliations:** ^1^Proteomics Research Center, Faculty of Paramedical Sciences, Shahid Beheshti University of Medical Sciences, Tehran, Iran; ^2^Gastroenterology and Liver Diseases Research Center, Research Institute for Gastroenterology and Liver Diseases, Shahid Beheshti University of Medical Sciences, Tehran, Iran

**Keywords:** Breast Neoplasms, Protein Interaction Maps, Algorithms, Gene Ontology

## Abstract

**Background:**

Management of breast cancer (BC) as a heterogeneous disease is very challenging. Biomarker discovery has been shown promising for this aim. Protein interaction mapping could provide further knowledge of the vital roles of these markers.

**Materials and Methods:**

Cytoscape and its plug-ins are used for network construction and evaluation. The plug-ins used in this study are STRING, Network Analyzer, GeneMANIA, and CluePedia.

**Results:**

The central proteins are enriched in transcription regulatory region DNA binding, regulatory region nucleic acid binding, regulatory region DNA binding, Fc receptor signaling pathway, cell cycle arrest, and immune response-regulating cell surface receptor signaling pathway.

**Conclusion:**

The introduced biomarkers and their related biological processes may show useful for the breast cancer diagnosis and monitoring; however, has to encounter more validation studies to be clinically applicable.

## Introduction

Breast cancer is the first cancer-related and the second cause of death in women [1]. Metastasis is the cause of death in breast cancer; in other words, if the tumor does not migrate to other parts of the body, it cannot be lethal [2]. The reports indicate that the incident of breast cancer in the western countries is higher than eastern countries due to the differential life style [1]. Breast cancer detection has been challenging and the first-line applied method for this aim is mammography which may not be applicable in all cases [3]. Early detection is the key for eradication of breast cancer and outcome improvements. Discovery of associated biomarkers in this stage could potentially introduce a solution in this regard. Many molecular studies including genomics, transcriptomics, and proteomics profiling have been conducted and were aimed to achieve a better understanding of underlying disease mechanisms [4, 5]. Ways of none invasion methods to detect the candidate biomarkers could be through serum, salivary, urine, tear, and breast fluids study [3]. Simultaneously, these molecular signatures could be the further analyzed by protein interaction mapping via topological examinations. Network medicine could accelerate recognition of the most crucial agents in terms of centrality properties. Thus, providing more validity of identified biomarkers of the condition that is considered for investigation [6]. In addition, these interactions can conduct the cell function in an organism with the unique role of central proteins [7]. Following this, enrichment analysis of the central proteins, provides further knowledge of the disease condition. To measure this, many algorithms are available either through Cytoscape or through online databases [8]. Network analysis, therefore, could identify multiple powerful therapeutic targets [9]. Since PPI network analysis is an analytical method to achieve various aims relative to molecular mechanism of diseases, there are several PPI network approaches to study breast cancer. In view of this fact, here, it is tired to identify the essential elements through PPI network analysis from the updated Breast cancer biomarkers rather than one or two conducted research for possible diagnosis and treatment purposes. In this regard, the recent findings related to breast cancer are screened to introduce some of the potent possible genes as a biomarker panel.

## Materials and Methods

The interactome landscape of breast cancer biomarkers was constructed through Cytoscape version: 3.0.6, which is an open interaction source for network construction [10]. The integrated database for network extraction is the string App (www.string-db.org) via evidence channels. STRING App provides platforms for network construction through four different queries including STRING, STITCH, DISEASES, and PubMed. String App searched the disease name “ Breast Cancer” and provided edge scores for each protein-protein associations in which here it is set to 0.5 [11].After network construction, for better understanding the topological features, Network Analyzer was used to determine nodes with highest scores of degree and betweenness centrality (BC).Nodes with highest scores of degree and betweenness values are called hub and bottlenecks. Those nodes with the co-existence of two parameters are known as hub-bottlenecks. To understand the characteristic of hub-bottlenecks, a sub-network of them was constructed via GeneMANIA to predict the function of the obtained genes. It is aimed to see if the genes are functionally related [12]. Two edge weighting in this network construction was based on pathway data and physical interactions. Genes participating in a same pathway are connected (the data is extracted from different databases including Reactome and BioCyc, via Pathway Commons). The interaction analysis is also queried from various sources such as BioGRID and Pathway Commons. The top hub-bottlenecks were considered as super hub-bottlenecks. The action type analysis of the core of super hub-bottlenecks was handled by CluePedia platform [13].CluePediaby setting cutoffs for three types of actions namely, expression, activation, and inhibition, analyzed the relationships. Different colors are set to indicate these interactions between genes and constructing a network of nested pathway. The source for action query is from STRING Action File in CluePedia Panel. Determination of these actions is through Kappa scoring which is a statistical method. The range of kappa score is from 0 to 1 which is customizable and can be shown by specifying thickness for the corresponding action type.

## Results

**Figure 1:**
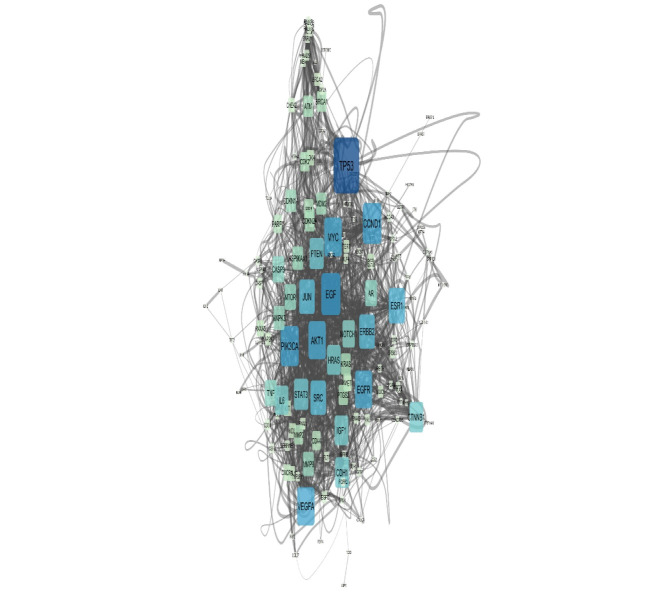
The main connected component of the queried network consisting of 156 nodes and 1495 edges is presented. The color cast and node size correspond to the degree value changes through the network.

**Figure 2:**
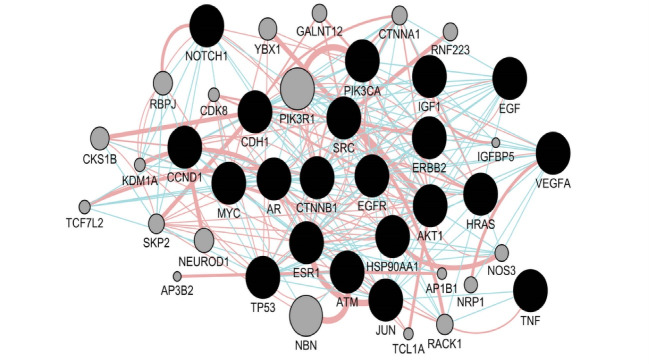
A network of identified central nodes with the 20 added neighbors (41 nodes and 155 links) visualized by GeneMANIA, a Cytoscape Plug-in is illustrated. The black colored nodes are the query ones while the gray ones are the surrounding nodes. The edge pink and blue colors imply on physical interactions and pathway relationship, respectively.

**Figure 3:**
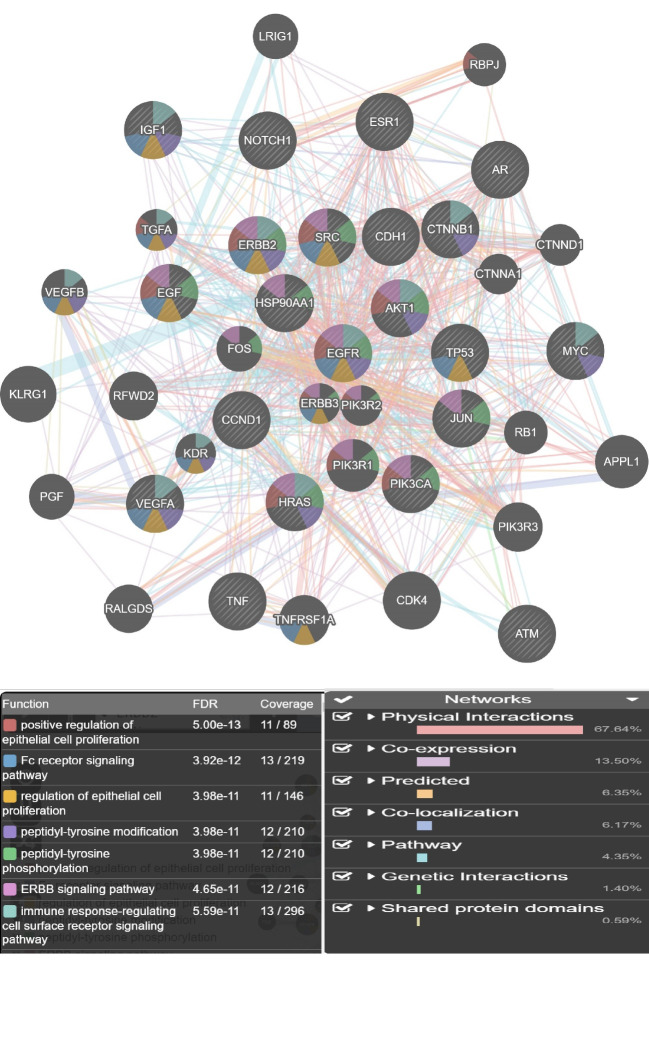
A: Zoomed view of interaction networkof hub-bottlenecks accompanied with the 20 added related nodes (the represented network in the figure 2) via GeneMANIA Online ** B:** Functions including positive regulation of epithelial cell proliferation, FC receptor signaling pathway, regulation of epithelial cell proliferation, peptidyl-tyrosine modification, peptidyl-tyrosine phosphorylation, ERBB signaling pathway, and immune response-regulating cell surface receptor signaling pathway and interaction properties of the network are shown.

**Figure 4:**
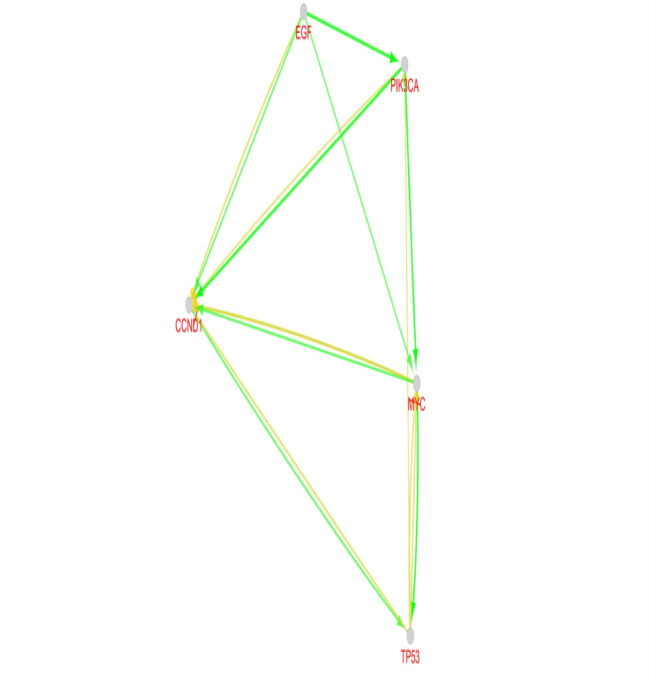
Nested pathway network of enriched super hub-bottlenecks (the top 5 nodes) from interaction enrichment=CluePedia String Actions, CluePedia. Green arrow indicate activation, yellow with round yellow end shows expression, and yellow with flat red end implies on inhibition action.

A network of 200 nodes related to breast cancer and 1497 edges considering cut 0ff of 0.5 was constructed via STRING DB. The network consists of 156 connecting nodes and 49 individuals. The main connected component of the queried network is shown in figure-1 with corresponding centrality parameters. After network construction, the centrality analysis showed some key nodes in the network that worth considering in depth understanding. These mentioned central elements that are 20% highest ranked nodes in degree and betweenness centrality were first analyzed and afterwards the common ones as hub-bottleneck nodes are tabulated in Table-1. For better understanding regarding physical and pathway interactions, the 21 central nodes and the 20 additional related proteins were included in a network (Figures-2 and 3). Furthermore, CluePedia explored the relationships between these agents considering expression, activation, and inhibition. This information provides further data related the identified central genes and their contribution in breast cancer risk. The critical finding about the top 5 hub-bottleneck nodes is presented in the figure-4.

## Discussion

Breast cancer is the most prevalent type of cancer in women worldwide while tremendous advances in diagnosis methods are made [14, 15]. Recent molecular studies have major impact on disease clarification and consequently helpful for treatment approaches. Prioritizing gene sets related to breast cancer as an interaction map was via String App, Cytoscape Plugin. Among these genes, some showed higher values in terms of centrality analysis knowing as hub-bottlenecks as it is clear from figure-1 and table-1. Additionally, the five top genes of the centrals are entitled as super hub-bottlenecks including TP53, EGF, CCND1, PIK3CA and MYC. These genes are repeatedly reported as novel biomarkers of different kinds of malignancies including gastric, colorectal cancer, hepatocellular cancer, ovarian cancer [16-21] and also breast cancer [21-23]. To focus on the central core relations and nature, the 21 central genes were selected for breast cancer sub-network creation via GeneMANIA as indicated in Figure-2. A condense interaction and pathway relations between these genes and their 20 neighbors is apparent. Furthermore, this core was more explored with regards to enrichment analysis. In Figure-3, the biological processes based annotation showed association of crucial terms for our central core, so these genes are functionally related. Seven crucial biological pathway were identified which among them positive regulation of epithelial cell proliferation and regulation of epithelial cell proliferation are the two important introduced processes. The role [24, 25] and their development is reported and discussed by several investigations. Peptidyl-tyrosine modification and peptidyl-tyrosine phosphorylation refer to role of EGFR in breast cancer [26]. EGFR is a hub-bottleneck node that is represented in the row 8 of Table-1. Role of FC receptor signaling pathway in immune system [27] and impact of immunological response in breast cancer are reported and investigated [28]. As it is shown in the figure-3 immune response-regulating cell surface receptor signaling pathway is involved in the biological processes of breast cancer. There is evidences that ERBB signaling pathway regulation is modified (via over expression process) in about 20% of breast cancer patients [29]. As mentioned above the five top hub-bottleneck nodes were identified as super central genes. Analysis indicates that all the determined biological processes are highlighted and associated to the super hub-bottlenecks. In addition, some terms are common between super hub-bottlenecks including PIK3CA, EGF, and TP53. In fact, the last two are involved in the same biological processes as regulation of epithelial cell proliferation and FC receptor signaling pathways. In this respect, EGF is involved in five biological processes that are common between biological processed of PIRK3CA and TP53. Therefore, as it is clear from here, EGF may have additional prominent role in breast cancer since its contribution in many processes. The malfunction of this agent may be resulted from deregulation of these biological processes. Further analysis of relationship between the super hub-bottlenecks is shown in figure-4. There are condense significant interactions between these central cores. Overexpression of CCND1 and MYC in cancer is investigated and confirmed [30, 31]. As it is depicted in the figure-4, TP53 as a tumor suppressor [32] inhibits expression of these two well-known oncogenes. Activation of CCND1 and MYC by EGF displays opposite effect relative to the role of P53. Since role of PIK3CA in tumor growth is essential [33] and EGF activates all oncogenes, it refers to the important impact of EGF in breast cancer. Regarding these actions, it can be concluded that EGF as the one of the important contributor to many biological processes, is also unique in super hub-bottlenecks action analysis. Meaning, EGF is the only super central gene that controls expression of the other oncogenes while no other genes have any effect on its expression. Consequently, these enriched terms may be important in breast cancer risk and potentially useful for disease screening.

## Conclusion

The identified genes as centrals may be more important in the diagnosis, progression, and treatment of breast cancer. However, more investigation regarding this claim is encouraged.

## Acknowledgment

This project was supported by Shahid Beheshti University of Medical Sciences (grant number: 14845).

## Conflict of Interest

There is no conflict of interest.

**Table 1:** The List of Hub-Bottlenecks Identified Through Topological Analysis Based on Degree and Betweenness Values Is Presented. The Nodes Are Sorted Based on Degree Value.

**Row**	**Display name**	**Degree**	**BC**
**1**	TP53	89	0.17
**2**	EGF	67	0.05
**3**	CCND1	65	0.05
**4**	PIK3CA	64	0.04
**5**	MYC	62	0.05
**6**	AKT1	61	0.04
**7**	VEGFA	61	0.02
**8**	EGFR	60	0.04
**9**	ESR1	57	0.05
**10**	ERBB2	55	0.03
**11**	JUN	55	0.03
**12**	SRC	55	0.02
**13**	CTNNB1	49	0.06
**14**	CDH1	49	0.01
**15**	HRAS	48	0.02
**16**	IGF1	48	0.02
**17**	NOTCH1	44	0.02
**18**	AR	42	0.03
**19**	TNF	42	0.01
**20**	HSP90AA1	38	0.01
**21**	ATM	35	0.03
